# Squamous Cell Carcinoma of the Skin and Coal Tar Creosote Exposure in a Railroad Worker

**DOI:** 10.1289/ehp.7373

**Published:** 2004-11-22

**Authors:** Chris Carlsten, Stephen Carl Hunt, Joel D. Kaufman

**Affiliations:** ^1^Department of Environmental and Occupational Health Sciences, and; ^2^Department of Medicine, Occupational and Environmental Medicine Program, University of Washington, Seattle, Washington, USA

**Keywords:** carcinoma, coal tar, creosote, environmental, exposure, occupational, railroad, squamous cell

## Abstract

A 50-year-old male railroad worker presented to his primary care physician with an erythematous, tender skin lesion on the right knee; a biopsy of this lesion revealed squamous cell carcinoma *in situ*. The site of the lesion was sun-protected but had been associated with 30 years of creosote-soaked clothing. In this article, we review dermal and other malignancies associated with creosote, along with creosote occupational exposures and exposure limits. This is a unique case, given the lack of other, potentially confounding, polyaromatic hydrocarbons and the sun-protected location of the lesion.

## Case Report

**Figure 1:**
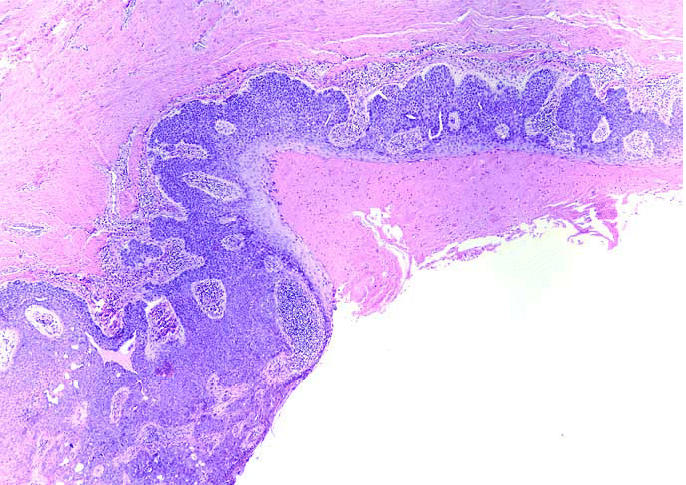
Hematoxylin and eosin stain, right knee skin biopsy. Magnification, 40×.

**Figure 2:**
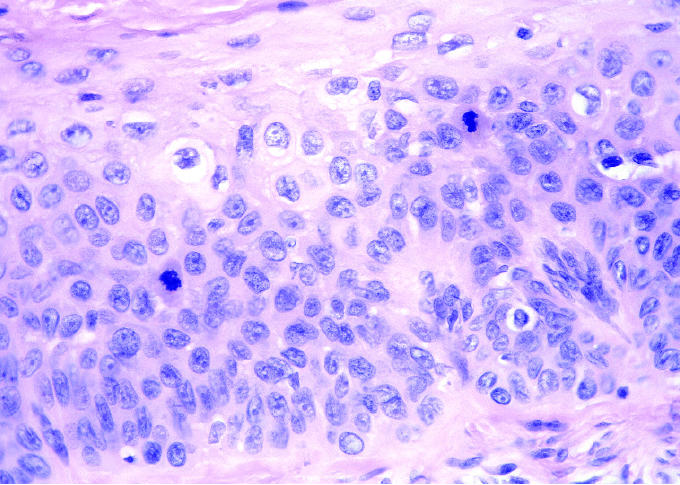
Hematoxylin and eosin stain, right knee skin biopsy. Magnification 100×.

A 50-year-old male railroad worker presented to his primary care physician with an erythematous, tender skin lesion on the anteromedial aspect of the right knee. The patient reported that the lesion had been present and intermittently tender over several years prior to presentation. The lesion was initially thought to be a simple cyst and was aspirated by the treating physician; antibiotics were also prescribed. The lesion did not resolve and was subsequently biopsied, revealing squamous cell carcinoma *in situ* (Figures 1 and 2).

The patient was referred to a surgeon; a definitive excision was performed, with subsequent completion of postoperative localized radiation therapy. The lesion did not recur.

The patient gave a history of having worked for a railroad company for 30 years before noting the skin lesion; most of this time had been spent on the “building and bridges” unit. This work involved the replacement of railroad track ties and the repair and construction of bridges and trestles, all of which involved the use of, and nearly daily handling of, coal tar creosote–treated ties and lumber. He reported that on a nearly daily basis, he carried, handled, kneeled on, or sat on this treated lumber. He also reported that the ties and timbers were heavily coated with creosote and that creosote commonly coated his work clothes at the end of his work shifts. He gave a history of wearing overalls, leather work-gloves, and long sleeves for a majority of his time at work. He stated that, despite wearing protective work clothes, he generally found creosote that had “filtered through” his clothing, particularly on his hands, wrists, and knees, and that he noticed this discoloration on his hands and knees at the end of most workdays. He was not aware of any other rashes or skin changes related to the exposures in these areas.

He had no history of other occupational exposures and reported having no significant sun exposure to the area of the lesion. He had no personal or family history of other skin disease or skin cancers. He was otherwise healthy, was a lifelong nonsmoker, and had no other significant medical history.

The physical examination revealed a healthy-appearing 50-year-old male with no medical concerns other than the skin lesion. Physical findings were unremarkable, with the exception of a well-healed 2-cm scar on the anteromedial aspect of the right knee (the focus of medial pressure upon kneeling). There were no other pertinent skin findings.

## Discussion

Polycyclic aromatic hydrocarbons (PAHs) were among the earliest work-related health hazards identified by contemporary occupational epidemiologic methods in the 18th century. Sir Percival Potts observed an apparent association between scrotal cancers and tar and soot exposures among chimney sweeps in London in 1775 (Harrison 2004). Although neither the precise chemical nature of the exposure nor a histologic analysis of the cancer is available, Potts’ report was ground-breaking.

Skin disease associated with creosote exposure was specifically reported as early as 1898, when MacKenzie published (in the *British Journal of Dermatology*) a case involving scrotal papillomatosis in a creosote worker (Mackenzie 1898). There have been limited subsequent case reports linking coal tar creosote with squamous cell cancers (Cookson 1924; Lenson 1956; Shimauchi et al. 2000). However, all of the lesions reported in these few cases were located in sun-exposed areas of skin, and it is possible that the lesions were secondary to the effects of sun rather than to creosote.

Animal studies have supported an exposure–disease link between creosote and skin cancers. Rous (1956) observed that study mice housed in creosote-impregnated wooden boxes developed surprising numbers of skin papillomas. Boutwell and Bosch (1958) documented skin carcinomas in mice exposed to creosote oils. In a human skin model, Schoket et al. (1988) observed that creosote induced adducts at levels thought to correlate with carcinogenicity in mice. In a cohort study involving 922 “creosote-exposed workers,” Karlehagen et al. (1992) found a standardized incidence ratio of 2.37 for skin cancer among exposed individuals compared with those not exposed, although no reliable data on individual exposure were available. As in prior case reports, sun exposure may have contributed to the elevated rates in the exposed group (Karlehagen et al. 1992).

PAHs result from the incomplete combustion of coal tar, pitch, coke, asphalt, and oil. Emitted as vapors of incomplete combustion, or pysolysis, PAHs precipitate as particles or condense onto soot particles. The term “creosote” is often used to describe these PAH-rich products of combustion and their distillates, and encompasses such products as wood creosote (from the combustion of beech and other woods), coal tar creosote (from the combustion of coal or coal tar), and coal tar pitch volatiles.

Coal tar creosote (containing over 300 different compounds, the majority of which are PAHs such as phenols, cresols, xylenols, pyridines, and benzene) is the most commonly used wood preservative in the United States [Agency for Toxic Substances and Disease Registry (ATSDR) 2002]. It is a thick, oily liquid that is amber or dark in color and is widely used in the treatment of telephone poles, railroad ties, marine pilings, and fence posts.

Occupational exposures to coal tar creosote are usually associated with work in wood preservation/pressure treatment facilities, fence building, bridge construction, utility work (telephone poles), aluminum smelting, and creosote site remediation. The ATSDR reported approximately 25,000 workers in nearly 100 wood treatment facilities using coal tar creosote in 1996 (ATSDR 2002). Additionally, nonoccupational exposures may result from the use of railroad ties in landscaping, burning of creosote-treated scrap lumber in fireplaces or woodstoves, and ingestion of contaminated groundwater (ATSDR 2004).

Non-cancer effects of coal tar creosote exposure involve primarily dermal and mucosal irritation manifested by dermatoses, photosensitivities, rhinitis, and conjunctivitis. The phototoxicity, in particular, is important because it compounds the irritative effects of coal tar creosote and presumably, therefore, increases its carcinogenicity (ATSDR 2002). Malignancies potentially associated with occupational coal tar pitch exposure include lung and prostate cancer in coke oven workers, lung cancer in foundry workers, lung and bladder cancer in aluminum smelter workers, and lung and stomach cancer in roofers (Harrison 2004). Other cancers suggested as being related to these compounds include renal cell carcinoma (Steineck et al. 1989), neuroblastoma (following parental occupational exposure) (Kerr et al. 2000), and non-Hodgkin lymphoma (Persson et al. 1989). The International Agency for Research on Cancer (IARC) has concluded that there is “sufficient” evidence for coal tar pitch (IARC 1998a) to be considered carcinogenic in humans, but that creosotes (IARC 1998b) have only “limited evidence” for human carcinogenicity, despite demonstrating “sufficient” evidence to establish carcinogenicity in animals.

Coal tar creosote exposures are regulated with an Occupational Safety and Health Administration (OSHA) permissible exposure limit (PEL) of 0.2 mg/m^3^ 8-hr time-weighted average (TWA; benzene soluble fraction; OSHA 1986) and a National Institute for Occupational Safety and Health (NIOSH) recommended exposure limit of 0.1 mg/m^3^ TWA (NIOSH 2002). Of note, there are no specific limits for dermal exposure. Nonetheless, engineering and personal protective equipment requirements established by a 1986 U.S. Environmental Protection Agency (EPA) “special review” (U.S. EPA 2000) are thought to have lowered exposures to levels below the established PEL. In the European Union, there are concerns that exposure limits may understate the carcinogenic risks of creosote exposure (Holme et al. 1999).

## Conclusion

In many occupational settings, workers are exposed to combinations of coal tar, coal tar pitch, and coal tar creosote, making it difficult to ascertain the mutagenic and carcinogenic risks associated with coal tar creosote exposure alone. There are, however, case reports as well as human and animal studies suggesting that creosote alone may be carcinogenic. The present case report, involving a railroad worker with a relatively “pure” long-term coal tar creosote exposure and a subsequent squamous cell carcinoma *in situ* in a non–sun-exposed skin area, further supports the relationship between coal tar creosote exposure and squamous cell carcinoma of the skin. Along with having such biological plausibility, it specifically eliminates the possibility of a primarily sun-related carcinogenesis. This case also emphasizes the value of a careful and detailed history in documenting the chronology, latency, circumstances, and relative dose characteristics that help establish causality is such cases.

Because there are significant numbers of individuals who have previously been exposed or are currently experiencing occupational and environmental exposures to coal tar creosote, the potential health effects of these exposures warrant attention. In cases for which substitutes for coal tar creosote is truly unfeasible, adoption of less permeable clothing may prevent such cases in the future.
